# Global Trends in Child Obesity: Are Figures Converging?

**DOI:** 10.3390/ijerph17249252

**Published:** 2020-12-10

**Authors:** María A. González-Álvarez, Angelina Lázaro-Alquézar, María Blanca Simón-Fernández

**Affiliations:** 1 Department of Economic Analysis, University of Zaragoza, 50005 Zaragoza, Spain; 2 Department of Applied Economics, University of Zaragoza, 50005 Zaragoza, Spain; alazaro@unizar.es (A.L.-A.); bsimon@unizar.es (M.B.S.-F.)

**Keywords:** BMI, child obesity, convergence, clubs, gender differences

## Abstract

Childhood obesity has become one of the most serious global health challenges of our time. The combined prevalence of overweight and obesity has rapidly increased worldwide during the last two decades, especially in some developing countries where obesity is reaching levels on a par with some industrialized countries, or even higher. This fast growth has occurred especially in countries in the midst of rapid social-economic transitions. Most international comparisons focus on the adult population while analyses focusing on the child population are more limited. Using the methodology developed by Phillips and Sul, this paper studies the worldwide evolution of children’s body mass index (BMI), overweight and obesity prevalence for a large sample of countries during the period 1975–2016. Our results indicate that the figures for BMI or the prevalence of obesity in different countries do not converge, while the opposite is the case for overweight prevalence in children. Furthermore, there is a non-linear relationship between obesity and income or human capital, indicating that low and middle-income countries require a strong initiative for health policies targeting obesity prevention.

## 1. Introduction

The United Nations Sustainable Development Goals (SDG) call on governments all over the world to act for universal and integrated changes to end hunger and malnutrition by 2030. Malnutrition includes various forms of undernutrition, but also includes overweight and obesity problems [1]. Although the term “nutrition” appears explicitly in Goal 2, malnutrition is an often invisible obstacle that affects many of the other SDGs since nutritional problems clearly affect the productivity of individuals which, in turn, can have negative implications for a country’s development.

In spite of this objective, in recent years new data have become available indicating that obesity has become one of the most serious global health challenges [2]. Traditionally, obesity has been considered an endemic disease in the most industrialized countries as a result of the concentration of the population in urban sites, the increase in consumption of processed foods, and more sedentary lifestyles. These overweight problems have important economic consequences. The study by Wang et al. [3] estimates the cost of obesity for national health systems, indicating that obesity is responsible for 2%–8% of health costs and 10%–13% of deaths in some parts of Europe, the latter figure being even worse in the USA where it may reach 17% by 2030. However, this problem has also increased significantly in many developing countries, where health systems are less prepared for the future complications arising from obesity [4,5,6]. Obesity prevalence has risen over the past two decades reaching levels similar to those in some industrialized countries, or even higher. It seems that the burden of obesity is shifting toward the poor from the rich countries [7].

Overweight and obesity in children and adolescents has become a problem of global concern that is increasingly attracting the attention of policymakers and researchers. Although current understanding of the health consequences of overweight and obesity is predominantly based on adult studies, evidence suggests that childhood obesity has a number of short-term and long-term health consequences. Obese children have a high probability of becoming obese adults, with higher risks of developing hypertension, diabetes, respiratory problems, certain cancers and other chronic diseases [8]. Childhood obesity could potentially result in the current generation of children having a shorter life expectancy than their parents because of its implications for adult mortality and morbidity [9,10,11,12]. Moreover, obese and overweight individuals have been reported to face higher levels of stigmatization, low self-esteem and a reduced health-related quality of life [13,14].

Substantial data have been collected in many countries allowing for the examination of time trends in obesity in adults and young people [2]. However, most international comparisons focus on the adult population [15,16,17], while studies of childhood obesity are often limited to individual country case studies [14,18,19,20,21,22]. This paper tries to fill this gap by analyzing convergence between a large set of countries, to determine whether there is indeed a closing of the gap between childhood obesity rates in industrialized and developing countries.

The objective of this article is, therefore, to analyze the evolution over time of overweight and obesity in children and adolescents in a wide range of countries using the methodology developed by Phillips and Sul [23,24], which allows us to determine if there is convergence for all countries and, if not, to determine if there is convergence between groups of countries. Although there are several works that analyze convergence using this methodology, they focus only on the adult population. Duncan and Toledo [25] and Kasman and Kasman [26] are worth mentioning in this regard. The first study analyses convergence of body mass index (BMI) in a sample of 172 countries, finding convergence only between groups of countries (three and six convergence clubs for adult female and male, respectively). It also analyzes the determinants that mark the composition of these clubs, including globalization, human capital, income, and rate of urbanization. On the other hand, Kasman and Kasman [26] expand the analysis of BMI to include an analysis of convergence in obesity and overweight rates in 35 OECD countries. They found that obesity rates follow the same speed of convergence; however, there is no evidence of convergence of BMI, for either male or female adults, or of overweight rates for females.

It should be noted that BMI has been the most widely used indicator to analyze convergence in overweight and obesity in different studies. However, using the average body mass index as the only comparative element between countries has some limitations. Two countries with the same average BMI could have very different overweight and obesity rates. In this context, we aim to determine whether a wide sample of countries converges at the same steady state using three different indicators for young children: BMI, overweight prevalence and obesity prevalence. We use a sample of 198 countries during the period 1975–2016 and explain the determinants that mark the convergence between countries.

The remainder of the paper is organized as follows. Section 2 describes the data, methodology and analysis framework. Section 3 presents the empirical results of the convergence analyses and a description of the clubs. Finally, Section 4 draws the most important conclusions and identifies the economic implications and policy recommendations.

## 2. Methodology

In addition to the traditional concepts of beta and sigma convergence, the concept of club convergence has attracted increasing interest in the literature. Baumol [27] originally introduced this notion to describe convergence among a subset of national units. Two distinct methodological strategies appear in the literature on the analysis of convergence clubs. The first arises from Quah [28,29], where the hypothesis of convergence clubs is tested based on the dynamics of the distribution of the variable of interest, using a combination of non-parametric methods such as stochastic kernel density functions and Markov chains. The second methodological strategy for the analysis of convergence clubs is derived from parametric models of beta convergence when assumptions of heterogeneity are included in the analysis. When differentiation or heterogeneity of individual economies is incorporated, it is possible to analyze whether economies tend to one or more common equilibria, in which case different convergence clubs are formed [30,31].

More recently, Phillips and Sul [23,24] proposed a time-varying factor model that allows for individual and transitional heterogeneity to identify convergence clubs. An interesting aspect of the analytical framework provided by Phillips and Sul [23] is that the rejection of the null hypothesis of convergence at the full panel level does not imply evidence against convergence at the subgroup level within the panel. This approach has several advantages [32]. First, it allows for different time paths as well as individual heterogeneity. Second, this methodology enables the endogenous determination of convergence clubs, unlike other approaches in which economies are grouped a priori. Third, the test is robust to heterogeneity and to the stationarity properties of the series. This overcomes some drawbacks of the standard methods because the presence of transitional heterogeneity invalidates the traditional unit root and cointegration approach. In addition, other critical points of the analysis of the partial correlation of the variable of interest (β-convergence) highlighted in [28,33] are solved.

In this section, we briefly describe the method developed by Phillips and Sul [23] (PS hereafter). We define $X_{it}$ as the variable under analysis (BMI, Overweight rate or Obesity rate), i and t being the indicators of country and time, respectively. We can decompose $X_{it}$ into the common component across countries ($\mu_{t}$) and the idiosyncratic component ($\delta_{it}$) as follows: $X_{it}=\delta_{it}\mu_{t}$. This methodology proposes an analysis of the time evolution of the idiosyncratic component; if $\delta_{it}$ converges towards δ, there is evidence in favor of the hypothesis of convergence. In order to remove the common factor, the relative transition component ($h_{it}$) and its cross-sectional variation ($H_{it}$) are defined:

(1) $$h_{it}=\frac{X_{it}}{N^{-1}\;\sum_{i=1}^{N}X_{it}}=\frac{\delta_{it}}{N^{-1}\;\sum_{i=1}^{N}\delta_{it}}$$

(2) $$H_{it}=N^{-1}\;\overset{N}{\underset{i=1}{\sum }}{\left(h_{it}-1\right)}^{2}\overset{as}{\to }0,\;as\;T\overset{as}{\to }\;\infty \;$$

In the presence of convergence, $h_{it}$ would tend to 1 and $H_{it}$ to 0 when time moves towards infinite. Formally, the hypothesis of convergence is tested by defining the log-t regression:

(3) $$log\frac{H_{1}}{H_{t}}-2log\left[\log\left(t\right)\right]=\alpha +\beta \log\left(t\right)+u_{t},\;t=\;\left[rT\right]+1,\;\ldots ,\;T$$

where r takes the value 0.3 according to the suggestion of [23] for this type of dataset. The null hypothesis of convergence (β=0) is rejected if the t-statistic takes values lower than −1.65.

As mentioned above, if the hypothesis of convergence is rejected, the PS methodology develops a clustering algorithm that groups countries that converge into the same steady state (we follow the proposal of [34], who slightly amends the PS algorithm, according to [35]). In order to extract the long-run trend and remove the short-run erratic behavior, following the recommendation of PS we have detrended $X_{it}$ by the use of the Hodrick and Prescott [36] filter. The smoothing parameter of the Hodrick-Prescott method can condition the results of the filtering, as noted in [37]. Following [24], we have defined λ=400. Results do not change if we change λ to 100, a standard value for annual data. The identification of convergence clubs is achieved through the application of an iterative algorithm that is summarized in Appendix A.

## 3. Data and Empirical Results

### 3.1. Data

The data are taken from Non-Communicable Diseases Risk Factor Collaboration (NCD-RisC), a network that collected worldwide trends in body-mass index, underweight, overweight, and obesity from 1975 to 2016 based on a pooled analysis of 2416 population-based measurement studies in 128 million children, adolescents, and adults. The database offers age-standardized values for mean BMI, overweight prevalence and obesity prevalence among children and adolescents aged 5 to 19 years. Cut-offs used to define overweight and obesity for children and adolescents vary by age and sex because of the natural growth in childhood and adolescence. An overweight child is defined as having a weight-for-age BMI ratio that is more than 1 to 2 standard deviation from the median (1SD < BMI < 2SD), while an obese child has a weight-for-age BMI ratio that is more than 2 standard deviation from the median (BMI > 2SD). The median is derived from an international reference population defined by the World Health Organization (WHO).

**Figure A1:**
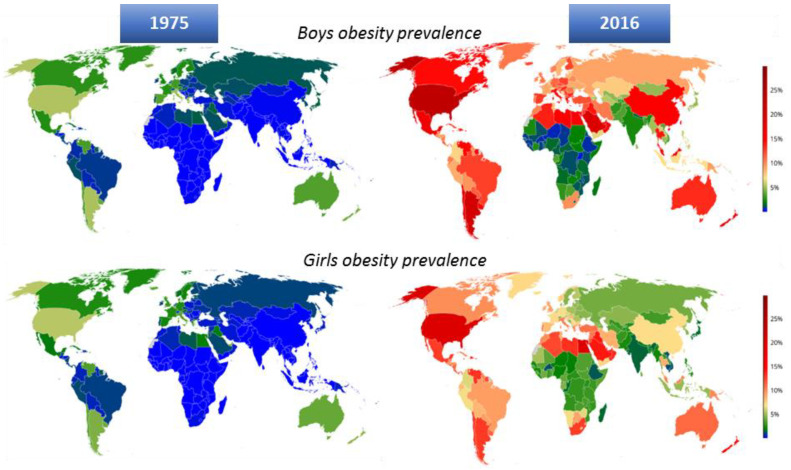
Average Obesity prevalence by club membership. Source: NCD-RisC [48].

Available data show that BMI and the prevalence of overweight and obesity is substantial in many countries around the world, but large variations exist. There are also considerable gender differences across regions and within them (see Figure A1 in Appendix B). Looking at mean values for the entire period (Table 1), the combined prevalence of overweight and obesity is high in western and industrialized countries, such as the USA, Canada and European countries, but Oceania is the region with the highest value in all three indicators. In Oceania, the obesity rate in 2016 doubled that of Europe for boys and tripled it for girls. On the other hand, Latin American countries, as a whole, exceed the total average both in terms of BMI and in overweight and obesity rates. This fact is especially marked in the case of girls, where the obesity rate exceeds the values reached in European countries due to the rapid increase experienced in the last two decades. In Africa and Asia, the combined prevalence of overweight and obesity is quite low, typically less than 5% or 10%. However, the prevalence has accelerated its pace of growth over the past two decades, especially in Africa.

**Figure A2:**
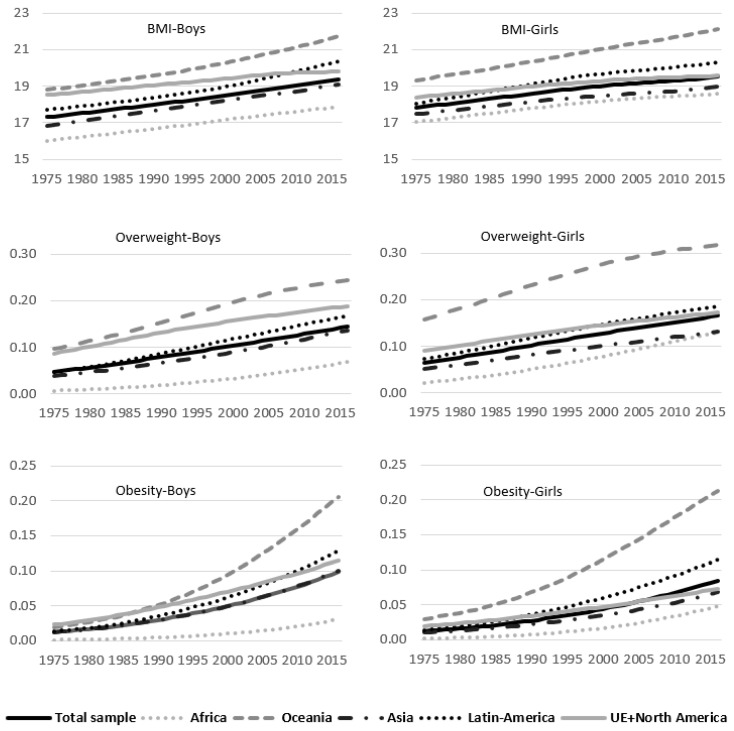
BMI, overweight and obesity evolution by gender and region.

**Figure A3:**
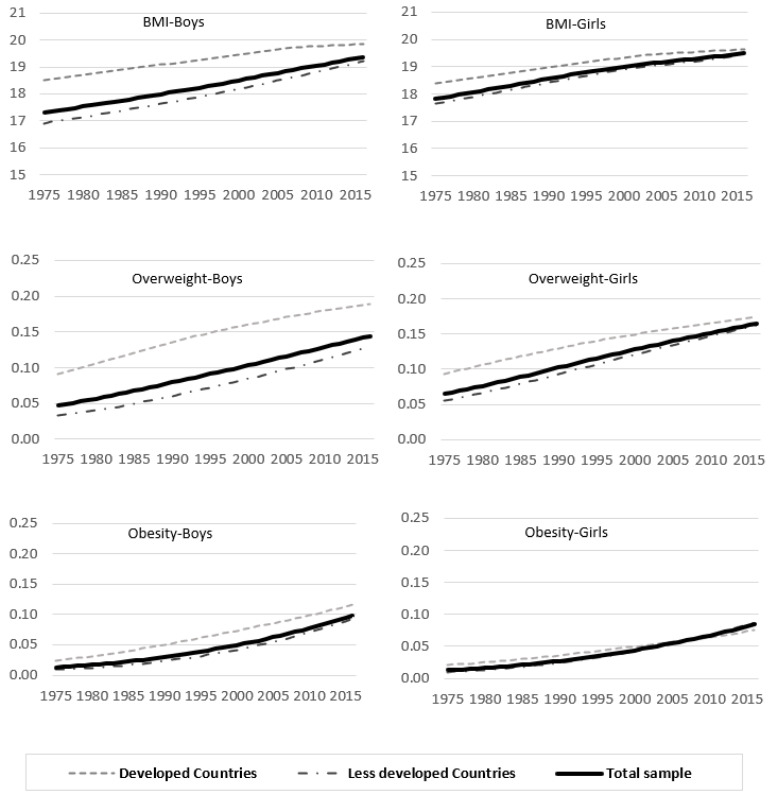
BMI, overweight and obesity evolution by gender and level of development.

Figure A2 and Figure A3 in Appendix C show the evolution over time of the three indicators by region and economic development, respectively. In some developing countries, children’s overweight and obesity has increased tremendously over the past two decades, with a combined prevalence within some regions reaching levels on a par with some industrialized countries, or even higher. This fast growth has occurred especially in countries that are in the midst of rapid social and economic transitions. If we focus on the last decade, nine out of the ten countries where obesity has increased most (for both girls and boys) are located in Oceania. For girls, the tenth country is South Africa, where obesity increased by eight percentage points, from 4.9% in 2007 to 12.8% in 2016. For boys, China is ranked seventh with respect to the increase in the obesity rate. China, in particular, is illustrative of dramatic increases in obesity and overweight prevalence that outpace rates observed in many industrialized countries. Its male obesity rate grew by 9 percentage points between 2007 and 2016, reaching 15.4% in 2016. The change for girls was smaller, 4 percentage points, with an obesity prevalence reaching 7.1% in 2016.

There are very few exceptions to this generally increasing trend. Japan, Denmark and the UK are the only three countries within the whole sample where obesity has experienced a slight decrease during the last decade (female obesity only, as male obesity has continued to grow). However, a decrease in the speed of growth is observed in most European and other industrialized countries such as Canada and USA.

Ranking countries by incidence of obesity in 2016, the first 13 countries with the highest rate of female obesity are located in Oceania, with rates fluctuating from 33.4% in Nauru to 22.3% in Micronesia. Kuwait, the first non-Oceanic country appearing in the ranking, has a female obesity rate of 20.2%, followed by the United States with an incidence of 19.5%. In the case of boys, the top positions in the ranking are also held by Oceania, with Nauru leading in first place with an obesity rate of 33.1%. Kuwait is in ninth position in the ranking (25.4%) and USA is placed in the 12th position (23.4%).

### 3.2. Results and Discussion

#### 3.2.1. Characterization of the Convergence Clubs

When applying the log t convergence test across 198 countries over the 1975–2016 period, the hypothesis of overall convergence is rejected at a 5% significance level for BMI and the obesity rate. This is not the case for the overweight rate. Hence, we can conclude that countries do not converge to the same equilibrium in terms of BMI or the prevalence of obesity, while they do converge in terms of overweight prevalence in children.

It seems appropriate to analyze the potential convergence processes in more homogenous groups by geographical regions and distinguishing between developed and less developed countries. The evidence against the null hypothesis in BMI and obesity prevalence is still overwhelming, especially with regard to the first indicator. In terms of degree of divergence, we find that this depends on the examined ratio: BMI shows that all regions and developed and underdeveloped countries diverge among themselves, while the obesity rate points to divergences in Africa, Oceania and developing countries for both male and females, and Asia also diverges in the case of males. As for overweight prevalence, most subgroups converge to the same long-run equilibrium, exhibiting a similar pattern of behavior. The exceptions are African and developing countries for boys and Oceania for girls.

The disparities reflected in Table 2 reinforce our main premise: convergence analysis results change depending on the indicator used and therefore using only BMI as an indicator of obesity can lead to erroneous conclusions. It is important to combine this information with other indicators to determine the real dimension of the problem and to be able to analyze the situation from a more comprehensive perspective. Besides, we can only compare our findings with those obtained in previous studies regarding the adult population, since there are no previous studies analyzing child obesity convergence. The overall conclusion is that the use of the PS methodology allows us to mostly reject the null hypothesis of convergence for children and adolescents, similar to the conclusions of previous papers on adults. Reference [25] found convergence among European countries, but not in the rest of the world. Reference [38] reject the null hypothesis of convergence for BMI growth rates among OECD countries. Reference [26] test convergence in BMI, obesity and overweight rates, also in OECD countries. They found that obesity rates are following the same speed of convergence; however, there is no evidence of convergence of BMI, for either male or female adults, or of overweight rates for females.

**Figure 1:**
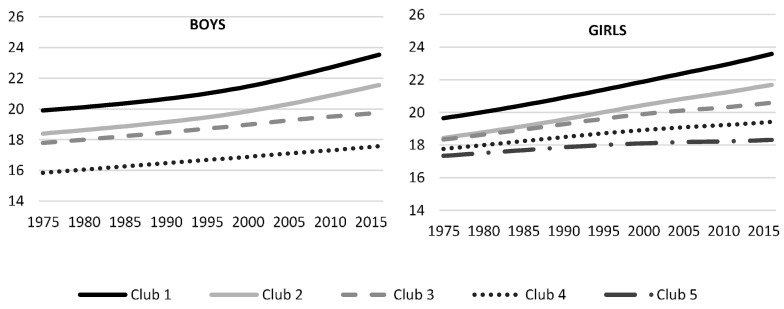
Average BMI by club membership.

**Figure 2:**
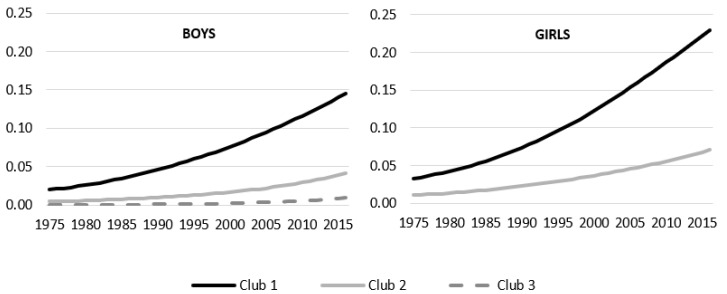
Average Obesity prevalence by club membership.

In order to identify some convergence clubs, we can apply the clustering algorithm designed by PS for the mean BMI and for the obesity rate. We illustrate the club membership in Figure 1 and Figure 2 for both indicators, and the list of countries included in each club is detailed in Appendix D for the BMI and Appendix E for the obesity rate. The clusters are ordered from higher to lower values of the indicator. Regarding BMI, there are four convergence clubs for boys and five convergence clubs for girls. Average values of BMI by Club appear in the first column of Table 3. For boys, Club 1 is made up of five countries, all located in Oceania, with an average BMI value of 21.31. Club 2 integrates 20 countries with a BMI of 19.68. This club concentrates six countries from Oceania, 4 from Asia and 10 from Latin America. Club 3, the most numerous with 114 countries and an average BMI of 18.76, includes all European countries (except Romania and The Netherlands, which are positioned in Club 4), most of the Asian countries (28) and Latin America (25), Canada and USA. Also, included in this club are 15 African countries and 5 oceanic countries. Finally, Club 5 has 57 countries mostly from Africa (36) and Asia (18). Two countries, Ethiopia and Senegal, do not converge in any of the previous clubs. Both countries have very low age-standardized mean BMI for both sexes, and lower for boys than for girls.

Figure 1 shows the evolution of BMI during 1975–2016. For boys, Club 1 exceeds the average of Club 2 by 1.5 points at the beginning of the sample period, although this gap is amplified to 2 points in 2016. It is also worth noting that, as time passes, Clubs 1 and 2 increase the distance from Clubs 3 and 4, with lower BMIs. The most industrialized countries are mostly concentrated in Club 3.

We find five convergence clubs for girls with 7, 9, 46, 58 and 78 countries, respectively. Appendix D reports the distribution of countries per world region and the complete list of countries in each club. Club 1 concentrates 7 Oceanic countries with the highest BMI (21.46 on average). The composition of this group is similar to the boys’ Club 1. Club 2, with an average value of 20.4 for BMI, has an equal number of countries from Oceania and Latin America. South Africa is also included in this club. Club 3 is formed mainly by Asian and Latin American countries, but it has a more heterogeneous composition with the presence of countries from Africa, Oceania, Europe and North America. Europe predominates in Club 4 (with a BMI value of 18.67), but also includes countries from Asia (14) and Africa (13). The most important region in Club 5 is Africa (35), followed by Asia (24) and Europe (16).

The dispersion among the five groups increases over time, as the clubs with the highest BMI are those that were growing the fastest. The distance between the first and the fifth club more than doubled during the 41 years of analysis, from 2.3 in 1975 to 5.4 in 2016.

Turning to obesity, three clubs are created for boys and two for girls. Club1 for boys is the most numerous, being composed of 113 countries with the greatest obesity problems. With respect to the geographical distribution, this Club includes most of the Oceanic countries (15 of the 17 that make up the sample) and Latin America (except Colombia, Peru and Saint Lucia), East and South Asian countries (22), 7 African countries (mostly from the North), 30 European countries, USA and Canada. Club 2, with an obesity rate of 1.6%, is mainly composed of African and Asian countries, although there is also a significant presence of European countries, especially from the north and east. Finally, Club 3 is composed of 12 countries from sub-Saharan Africa.

From 1975 to 2016 obesity prevalence increased in every country of the sample. The incidence of obesity increased very notably in Club 1 over the years, widening the gap with the other two clubs at the end of the period. Club 2, which began with a small incidence of 0.4% in 1975, reached 4% at the end of the period. It is also observed that the speed accelerated in the last two decades in all three clubs.

Finally, the results for obesity in girls classifies countries into two clubs according to their obesity prevalence. Club 1 consists of 17 countries with an obesity rate of 11% and Club 2 includes 181 countries with an obesity rate of 3.4%. The lower number of countries in Club 1, with the highest obesity rate, and the high concentration of countries in Club 2 may be related to the greater pressure women feel towards certain physical stereotypes, leading to greater homogenization towards an ideal body weight [39]. This could lead to a greater concentration among girls in terms of the number of convergence groups as occurs with adult women [25].

The gap between the two groups increased from 2 percentage points in 1975 to 16 points in 2016. In the first group there is an important presence of oceanic countries (11), but it also includes four African countries (Botswana, Egypt, Lesotho and South Africa), Kuwait and USA.

#### 3.2.2. Factors Driving Club Formation

Several factors can explain the differences between countries and regions. For this analysis, we have used those that commonly appear in the literature as risk factors associated with obesity. Duncan and Toledo [25] include a good review of these aspects. We include the following determinants in our analysis:

- GDP per capita based on purchasing power parity (PPP) converted to international dollars using purchasing power parity rates. Data are in constant 2017 international dollars. Source: World Development Indicators-World Bank [40].
- Two governance indicators: Political Stability and Voice. Political Stability and Absence of Violence/Terrorism measures perceptions of the likelihood of political instability and/or politically motivated violence, including terrorism. Voice and Accountability captures perceptions of the extent to which a country’s citizens are able to participate in selecting their government, as well as freedom of expression, freedom of association, and a free media. Scores range from −2.5 to 2.5 in both indicators. Source: World Development Indicators-World Bank [40].
- Human Capital Index (HCI) measures the amount of human capital that a child born today can expect to attain by age 18, given the risks of poor health and poor education that prevail in the country where she lives. The index score ranges from zero to one and measures the productivity as a future worker of a child born today relative to the benchmark of full health and complete education. Source: World Development Indicators-World Bank [40].
- Urban rate is the percentage of population living in urban areas as defined by national statistical offices. Source: World Development Indicators-World Bank [40].
- Globalization Index measures the economic, social and political dimensions of globalization. The globalization Index comes from [41].

Table 3 presents the indicators that characterize the convergence clubs. Aside from the mean BMI and obesity rate in 2016, we report the mean of each indicator for every club. For boys and girls, we observe that the relationship between BMI and GDP is nonlinear. For boys, GDP grows between Club 1 and 3, but decreases significantly in Club 4, which has the lowest BMI. For girls, the highest value of GDP is reached in Club 3, and then falls in Club 4 and Club 5. This indicates that as the GDP grows, the average BMI also grows. However, there comes a point where the relationship between the two variables becomes negative, and from that moment on BMI starts to fall while GDP continues to grow. The human capital index and the urbanization rate follow a similar pattern. This holds also for globalization in the case of boys, but not for girls.

Interestingly, the previous characterization does not hold for the obesity clubs. For boys, there is a direct relationship between GDP and the obesity rate. The higher the economic well-being, the higher the obesity rate. The same occurs with respect to globalization, the rate of urbanization and the human capital index. For girls’ clubs the opposite is true. Club 1, with a higher rate of obesity, has a lower GDP, lower level of globalization and also lower HCI.

To estimate the effects of the covariates on BMI and the obesity rate, we estimate an ordered logit model for each measure, since the dependent variables are ordinal and ranked in descending order according to the different steady states. Apart from the factors previously described, we have also incorporated dummy variables to capture geographical effects. The results obtained for BMI and the obesity prevalence are presented in Table 4.

The ordered logit estimation indicates that the effect of GDP on BMI is negative. However, the square of this variable has a positive value, reinforcing the previous idea that there is a nonlinear relationship between income and BMI. This indicates that, as countries increase their income level, BMI grows, but there comes a moment when the relationship is reversed. This effect also occurs with respect to the rate of obesity, with those countries with higher incomes being those that manage to start reducing child obesity problems. This result is in line with recent studies analyzing the existence of an obesity Kuznets curve, describing an inverted U-shaped relationship between the two variables [42,43,44]. The idea behind this is that, as incomes rise, resources become available to buy more food and obesity rates increase. However, as income continues to rise, personal health becomes a more valued asset and people shift their consumption to healthier foods, which eventually reduces obesity rates [43].

The relationship with respect to human capital is similar. Usually, higher human capital is associated with lower overweight problems [25]. However, some studies in developing countries find that a higher level of parental education is associated with a greater risk of children becoming overweight or obese [14]. In our case, a non-linear relationship is observed in which higher levels of human capital are associated with a greater risk of obesity, but this relationship is reversed at a point where obesity begins to decline.

Governance variables tend to have a direct impact, but only for boys, and not girls. Some studies suggest that rapid urbanization is the cause of major changes in eating patterns and physical activity levels that tend to increase the risks of obesity in children. Highly urbanized areas have resulted in less access to sports activities and other means of physical exercise [45,46]. In our case, the percentage of population living in urban areas is positive, as expected, but it is only significant for boys: higher population concentration in urban areas implies higher BMI. Finally, globalization, which appears as a significant factor to explain the increase in obesity rates in many previous studies of adult populations [47], does not seem to have a significant effect on childhood obesity.

## 4. Conclusions

The combined prevalence of overweight and obesity has tripled in many countries worldwide since the 1980s and the number of people affected is expected to continue to rise. Substantial data have been gathered in many countries allowing for the examination of time trends in obesity in adult and young people. However, most international comparisons focus on the adult population while analyses focusing on the child population are more limited.

In general, the combined prevalence of overweight and obesity in children is much higher in developed countries than in developing ones. However, the incidence of obesity has accelerated in many developing economies, especially in Oceania or Latin America, reaching rates above those found in the USA. In addition, overweight problems are more prevalent in developing countries among girls, while in developed countries they are more severe among boys. In Africa and Asia, where the combined prevalence of overweight and obesity is quite low, the prevalence has accelerated its pace of growth over the past two decades, especially in Africa. However, a decrease in the speed of growth is observed in most European and other industrialized countries such as Canada and USA. The only exceptions to this generally increasing trend are Japan, Denmark and the UK, where obesity has experienced a slight decrease during the last decade. The reasons for this relative improvement in the most developed countries may be related to a greater effort by their governments to combat the main causes of child obesity, by establishing special taxes on foods rich in fats and sugars, as well as launching campaigns to raise awareness of healthy eating.

This article focuses on analyzing whether there is convergence between countries, to determine if indeed there is a closing of the gap in the prevalence of childhood obesity. The analysis, using the methodology developed by Phillips and Sul [23,24], determines that countries are converging in terms of child overweight, but rejects the convergence hypothesis for BMI and the obesity rate. In a second step, a number of clubs is identified. Those countries that converge to their high-obesity counterparts within unhealthy clubs should be more concerned about the adverse effects on average body weights, especially if they are developing countries. Although there are some differences between boys and girls, the economic development of the countries and the human capital index have a clear effect on the composition of the clubs in either case. This effect is not linear, but as countries advance, both economically and in their human capital, both the BMI and the childhood obesity rate increase. However, when a certain level of development is reached, this effect is reversed and greater control over childhood obesity rates begins to be achieved. Therefore, our analysis supports the argument that low and middle-income countries, many of which are currently enjoying high economic growth, require a strong initiative for health policies targeting obesity prevention.

Considering the consequences that overweight and obesity have on the child population, it is necessary to implement measures that help control the rapid growth that this phenomenon has been experiencing in recent decades. Even more worrying is the fact that the rapid expansion is occurring mainly in developing countries, since a slowdown in the growth of child obesity rates is observed in the more industrialized economies. In some developing countries, there are simultaneously high rates of undernutrition and obesity. These countries often receive international aid only focused on malnutrition but not targeting obesity, so programs should be designed in a manner which responds to this problem. A better understanding of the childhood obesity epidemic will help guide public intervention efforts and develop effective programs and policies.

Obesity has significant health and financial implications for countries, which may make it difficult to achieve the SDGs by 2030. Therefore, preventing childhood obesity should be a high priority on national policy agendas in many countries. Work is needed especially on prevention measures that avoid young people from gaining more weight than is considered healthy. Risk factors, educational interventions, and changes in attitudes need to be identified that can be effective in reducing risk across the population as a whole, and especially among young people. Countries should improve the welfare of individuals and human capital to encourage healthy lifestyles and reduce obesity risks.

## Figures and Tables

**Table 1:** Overall sample means by gender and regions: 1975–2016.

	Boys			Girls		
	BMI	Overweight	Obesity	BMI	Overweight	Obesity
Total sample	18.293	0.093	0.045	18.746	0.116	0.040
Africa	16.942	0.030	0.010	17.927	0.069	0.017
Oceania	20.059	0.175	0.087	20.692	0.250	0.102
Asia	17.946	0.082	0.045	18.271	0.092	0.032
Latin America	18.814	0.105	0.057	19.307	0.132	0.054
EU & North America	19.232	0.143	0.063	19.086	0.135	0.043
Developing Countries	17.975	0.076	0.039	18.623	0.108	0.039
Developed Countries	19.263	0.146	0.065	19.119	0.139	0.045

**Table 2:** Estimated parameters and t-test statistics of the convergence test.

	Boys		Girls	
	Beta	T-Stat	Beta	T-Stat
Body Mass Index				
Total sample	−0.736	−99.27	−0.954	−126.43
Africa	−0.912	−289.28	−0.939	−70.54
Oceania	−1.178	−100.52	−1.905	−27.30
Asia	−0.741	−95.06	−0.636	−119.15
Latin America	−0.739	−36.76	−1.067	−82.54
EU & North America	−0.882	−22.10	−0.782	−24.91
Developing Countries	−1.045	−31.65	−1.003	−123.60
Developed Countries	−0.953	−127.85	−1.007	−41.35
Prevalence of Overweight				
Total sample	0.032	0.98	0.229	6.09
Africa	−0.264	−8.74	0.284	4.95
Oceania	0.259	7.58	−0.032	−3.52
Asia	0.388	8.50	0.455	9.78
Latin America	1.261	15.66	0.714	10.95
EU & North America	0.719	9.69	0.558	12.55
Developing Countries	−0.151	−5.65	0.082	2.35
Developed Countries	0.580	9.36	0.422	10.67
Prevalence of Obesity				
Total sample	−0.529	−26.85	−0.239	−8.51
Africa	−0.765	−65.57	−0.714	−53.82
Oceania	−1.089	−129.16	−0.858	−93.07
Asia	−0.283	−9.35	−0.043	−1.28
Latin America	−0.018	−0.36	0.353	5.49
EU & North America	0.311	5.70	0.205	5.91
Developing Countries	−0.723	−53.38	−0.450	−21.43
Developed Countries	0.191	3.83	−0.018	−0.73

**Table 3:** Mean values of descriptive variables by Cluster.

Body Mass Index							
	BMI	GDP	Political Stability	Voice	HCI	Urban Rate	Globalization
Boys							
Club 1	21.31	12,406.0	1.02	0.93	0.51	62.73	47.99
Club 2	19.68	24,751.1	0.57	0.35	0.57	65.18	57.97
Club 3	18.76	25,345.3	0.06	0.13	0.64	67.04	68.78
Club 4	16.70	7122.8	−0.53	−0.49	0.45	41.60	52.81
Girls							
Club 1	21.46	8766.9	1.13	0.93	0.53	55.04	49.70
Club 2	20.04	19,672.8	0.45	0.80	0.59	75.94	64.00
Club 3	19.55	25,483.4	0.19	0.10	0.59	67.09	62.99
Club 4	18.67	22,347.0	0.04	0.16	0.62	61.76	67.20
Club 5	17.93	14,558.5	−0.40	−0.39	0.52	50.61	59.35
*Obesity Prevalence*							
	Obesity	GDP	Pol Stabil	Voice	HCI	Urban rate	Globalization
Boys							
Club 1	0.069	26,887.8	0.22	0.22	0.63	68.35	67.38
Club 2	0.016	12,044.9	−0.38	−0.32	0.52	48.87	58.01
Club 3	0.002	2147.9	−0.66	−0.48	0.37	34.45	49.04
Girls							
Club 1	0.112	15,200.0	0.53	0.56	0.51	60.42	57.32
Club 2	0.034	19,904.9	−0.10	−0.07	0.57	58.89	63.01

**Table 4:** Estimation (Ordered logit).

	Body Mass Index						Obesity Rate					
	Boys			Girls			Boys			Girls		
	Coefficient		t-Ratio	Coefficient		t-Ratio	Coefficient		t-Ratio	Coefficient		t-Ratio
GDP	−0.153	**	−2.83	−0.17	***	−4.48	−0.205	***	−2.94	−0.522	**	−2.26
GDP^2^	0.001	**	2.03	0.001	***	4.09	0.001	***	2.82	0.003	**	2.36
Polit. Stability	0.47	*	1.64	0.188		0.61	0.132		0.38	1.212		1.03
Voice	0.751	**	2.01	0.404		1.02	0.731	*	1.63	−2.209		−1.65
Human Capital Index (HCI)	−55.369	***	−3.34	−22.573	*	−1.75	−43.13	***	−3.22	−90.137	**	−2.00
HCI^2^	48.167	***	3.49	21.79	**	2.14	40.528	***	3.50	107.004	***	2.50
Urban rate	−0.027	*	−1.89	−0.003		−0.26	−0.017		−1.16	0.022		0.59
Globalization	0.011		0.25	−0.006		−0.14	−0.019		−0.50	0.075		0.79
Regions:												
Oceania	−5.396	**	−2.08	−4.13	***	−2.93	−4.373	***	−3.84	−1.715		−1.04
Asia	0.599		0.94	1.003		1.37	−0.71		−0.96	5.591		1.36
Latin Am.	−3.324	***	−3.27	−2.014	***	−2.70	−3.237	***	−3.13	20.401	***	9.14
EU & NA	−1.17		−1.21	1.03		1.19	−0.581		−0.62	2.246		1.75
Not observed	147			149			149			149		
R^2^-adjusted	0.43			0.22			0.39			0.45		
Log-ratio	−71.77			−141.32			−81.92			−17.04		

* Denotes statistical significance at the 10% level, ** at the 5% level, and *** at the 1% level.

## Appendix A

The identification of convergence clubs is done through the application of an iterative algorithm that is summarized in four steps: (1) observations are ordered with respect to the last-period value of the time series. For example, in the case of BMI, we order the countries in a descending order with the first country having the highest last-period BMI, the second with the next highest BMI and so on. (2) Form all possible groups (C_k_) by selecting the first k highest countries, with k = 2, 3, ..., N. Then, test for convergence using the log t test within each subgroup of size k. Finally, define the core club as the club for which the maximum computed log t statistic occurs, given that the log t statistics supports the convergence hypothesis. (3) From the remaining countries, add one country at a time to the core club and test for convergence through the log t test. Find all countries that, according to the log t test, converge to the same steady state with the core group. (4) Then, for the remaining countries, if any, repeat the procedure described in steps 1 through 3 to determine the next convergence club. Finally, terminate the procedure when the remaining economies fail to converge.

## Appendix D

### Appendix D.1. Body Mass Index Club Composition

**Table A1:** Number of countries by Club and region.

	Africa	Oceania	Asia	Latin	EU & NA	Total
**Boys**						
Club 1	0	5	0	0	0	5
Club 2	0	6	4	9	1	20
Club 3	15	5	28	25	41	114
Club 4	36	1	18	0	2	57
**Girls**						
Club 1	0	7	0	0	0	7
Club 2	1	4	0	4	0	9
Club 3	4	3	12	19	8	46
Club 4	13	2	14	9	20	58
Club 5	35	1	24	2	16	78

### Appendix D.2. BMI Club Composition for Boys

***Club 1 (5 countries):***

American Samoa, Palau, French Polynesia, Tokelau, Tonga.

***Club 2 (20 countries):***

United Arab Emirates, Antigua and Barbuda, Bahamas, Belize, Bermuda, Barbados, Chile, Micronesia (Federated States of), Haiti, Jamaica, Kiribati, Saint Kitts and Nevis, Kuwait, Marshall Islands, Nauru, Puerto Rico, Qatar, Saudi Arabia, Tuvalu, Samoa.

***Club 3 (114 countries):***

Albania, Andorra, Argentina, Armenia, Australia, Austria, Azerbaijan, Belgium, Benin, Bulgaria, Bahrain, Bosnia and Herzegovina, Belarus, Bolivia, Brazil, Brunei Darussalam, Canada, Switzerland, China, Cote d’Ivoire, Colombia, Costa Rica, Cuba, Cyprus, Czech Republic, Germany, Djibouti, Dominica, Denmark, Dominican Republic, Algeria, Ecuador, Egypt, Spain, Estonia, Finland, Fiji, France, Gabon, United Kingdom, Georgia, Greece, Grenada, Greenland, Guatemala, Guyana, China (Hong Kong SAR), Honduras, Croatia, Hungary, Ireland, Iran, Iraq, Iceland, Israel, Italy, Jordan, Kyrgyzstan, South Korea, Lebanon, Libya, Saint Lucia, Lithuania, Luxembourg, Latvia, Moldova, Maldives, Mexico, Macedonia (TFYR), Malta, Montenegro, Mozambique, Mauritius, Malaysia, Nicaragua, Norway, New Zealand, Oman, Panama, Peru, Papua New Guinea, Poland, North Korea, Portugal, Paraguay, Occupied Palestinian Territory, Russian Federation, Sudan, Singapore, Solomon Islands, El Salvador, Serbia, Sao Tome and Principe, Suriname, Slovakia, Slovenia, Sweden, Swaziland, Seychelles, Syrian Arab Republic, Thailand, Tajikistan, Turkmenistan, Trinidad and Tobago, Tunisia, Turkey, Taiwan, Ukraine, Uruguay, United States of America, Uzbekistan, Saint Vincent and the Grenadines, Venezuela, South Africa.

***Club 4 (57 countries):***

Afghanistan, Angola, Burundi, Burkina Faso, Bangladesh, Bhutan, Botswana, Central African Republic, Cameroon, DR Congo, Congo, Comoros, Cabo Verde, Eritrea, Ghana, Guinea, Gambia, Guinea Bissau, Equatorial Guinea, Indonesia, India, Japan, Kazakhstan, Kenya, Cambodia, Lao PDR, Liberia, Sri Lanka, Lesotho, Morocco, Madagascar, Mali, Myanmar, Mongolia, Mauritania, Malawi, Namibia, Niger, Nigeria, The Netherlands, Nepal, Pakistan, Philippines, Romania, Rwanda, Sierra Leone, Somalia, Chad, Togo, Timor-Leste, Tanzania, Uganda, Viet Nam, Vanuatu, Yemen, Zambia, Zimbabwe.

***Non-convergent Group (2 countries):***

Ethiopia, Senegal.

### Appendix D.3. BMI Club Composition for Girls

***Club 1 (7 countries):***

American Samoa, Palau, French Polynesia, Tokelau, Tonga, Tuvalu, Samoa.

***Club 2 (9 countries):***

Bahamas, Chile, Kiribati, Mexico, Marshall Islands, Nauru, New Zealand, Trinidad and Tobago, South Africa.

***Club 3 (46 countries):***

Andorra, United Arab Emirates, Antigua and Barbuda, Australia, Benin, Bahrain, Belize, Bermuda, Bolivia, Brazil, Barbados, Brunei Darussalam, Bhutan, Costa Rica, Dominica, Algeria, Ecuador, Egypt, Micronesia (Federated States of), United Kingdom, Greece, Grenada, Greenland, Guatemala, Iraq, Israel, Italy, Jamaica, Saint Kitts and Nevis, Kuwait, Malta, Malaysia, Nicaragua, Oman, Panama, Papua New Guinea, Puerto Rico, Paraguay, Occupied Palestinian Territory, Qatar, Saudi Arabia, El Salvador, Swaziland, United States of America, Saint Vincent and the Grenadines, Venezuela.

***Club 4 (58 countries):***

Afghanistan, Albania, Argentina, Austria, Belgium, Burkina Faso, Bulgaria, Canada, China, Colombia, Comoros, Cabo Verde, Cyprus, Germany, Dominican Republic, Spain, Estonia, Finland, Fiji, France, Gabon, Guyana, Honduras, Croatia, Hungary, Ireland, Iran, Iceland, Jordan, South Korea, Libya, Saint Lucia, Lesotho, Morocco, Maldives, Macedonia (TFYR), Mauritania, Nepal, Peru, Poland, Portugal, Romania, Rwanda, Singapore, Solomon Islands, Serbia, Sao Tome and Principe, Suriname, Slovenia, Seychelles, Syrian Arab Republic, Togo, Thailand, Tunisia, Turkey, Taiwan, Uruguay, Uzbekistan.

***Club 5 (78 countries):***

Angola, Armenia, Azerbaijan, Burundi, Bangladesh, Bosnia and Herzegovina, Belarus, Botswana, Central African Republic, Switzerland, Cote d’Ivoire, Cameroon, DR Congo, Congo, Cuba, Czech Republic, Djibouti, Denmark, Eritrea, Ethiopia, Georgia, Ghana, Guinea, Gambia, Guinea Bissau, Equatorial Guinea, China (Hong Kong SAR), Haiti, Indonesia, India, Japan, Kazakhstan, Kenya, Kyrgyzstan, Cambodia, Lao PDR, Lebanon, Liberia, Sri Lanka, Lithuania, Luxembourg, Latvia, Moldova, Madagascar, Mali, Myanmar, Montenegro, Mongolia, Mozambique, Mauritius, Malawi, Namibia, Niger, Nigeria, The Netherlands, Norway, Pakistan, Philippines, North Korea, Russian Federation, Sudan, Senegal, Sierra Leone, Somalia, Slovakia, Sweden, Chad, Tajikistan, Turkmenistan, Timor-Leste, Tanzania, Uganda, Ukraine, Viet Nam, Vanuatu, Yemen, Zambia, Zimbabwe.

## Appendix E

### Appendix E.1. Obesity Prevalence Club Composition

**Table A2:** Number of countries by Club and region.

	Africa	Oceania	Asia	Latin	EU & NA	Total
**Boys**						
Club 1	7	15	28	31	32	113
Club 2	34	2	22	3	12	73
Club 3	12	0	0	0	0	12
**Girls**						
Club 1	4	11	1	0	1	17
Club 2	49	6	49	34	43	181

### Appendix E.2. Obesity Club Composition for Boys

***Club 1 (113 countries):***

Albania, Andorra, United Arab Emirates, Argentina, American Samoa, Antigua and Barbuda, Australia, Austria, Bulgaria, Bahrain, Bahamas, Belarus, Belize, Bermuda, Bolivia, Brazil, Barbados, Brunei Darussalam, Canada, Chile, China, Costa Rica, Cuba, Cyprus, Czech Republic, Germany, Dominica, Denmark, Dominican Republic, Algeria, Ecuador, Egypt, Spain, Finland, Fiji, Micronesia (Federated States of), United Kingdom, Greece, Grenada, Greenland, Guatemala, Guyana, China (Hong Kong SAR), Honduras, Croatia, Haiti, Hungary, Indonesia, Ireland, Iran, Iraq, Iceland, Israel, Italy, Jamaica, Jordan, Kiribati, Saint Kitts and Nevis, South Korea, Kuwait, Lao PDR, Lebanon, Libya, Luxembourg, Morocco, Maldives, Mexico, Marshall Islands, Macedonia (TFYR), Malta, Montenegro, Malaysia, Nicaragua, Norway, Nauru, New Zealand, Oman, Panama, Palau, Papua New Guinea, Poland, Puerto Rico, North Korea, Portugal, Paraguay, Occupied Palestinian Territory, French Polynesia, Qatar, Romania, Saudi Arabia, Singapore, El Salvador, Serbia, Suriname, Slovakia, Slovenia, Seychelles, Syrian Arab Republic, Thailand, Tokelau, Tonga, Trinidad and Tobago, Tunisia, Turkey, Tuvalu, Taiwan, Uruguay, United States of America, Saint Vincent and the Grenadines, Venezuela, Samoa, Yemen, South Africa.

***Club 2 (73 countries):***

Afghanistan, Angola, Armenia, Azerbaijan, Burundi, Belgium, Burkina Faso, Bangladesh, Bosnia and Herzegovina, Bhutan, Botswana, Switzerland, Cote d’Ivoire, Cameroon, DR Congo, Congo, Colombia, Comoros, Cabo Verde, Djibouti, Eritrea, Estonia, France, Gabon, Georgia, Gambia, Guinea Bissau, Equatorial Guinea, India, Japan, Kazakhstan, Kenya, Kyrgyzstan, Cambodia, Saint Lucia, Sri Lanka, Lesotho, Lithuania, Latvia, Moldova, Madagascar, Mali, Myanmar, Mongolia, Mozambique, Mauritania, Mauritius, Malawi, Namibia, Nigeria, The Netherlands, Nepal, Pakistan, Peru, Philippines, Russian Federation, Sudan, Solomon Islands, Somalia, Sao Tome and Principe, Sweden, Swaziland, Togo, Tajikistan, Turkmenistan, Timor-Leste, Tanzania, Ukraine, Uzbekistan, Viet Nam, Vanuatu, Zambia, Zimbabwe.

***Club 3 (12 countries):***

Benin, Central African Republic, Ethiopia, Ghana, Guinea, Liberia, Niger, Rwanda, Senegal, Sierra Leone, Chad, Uganda.

### Appendix E.3. Obesity Club Composition for Girls

***Club 1 (17 countries):***

American Samoa, Botswana, Egypt, Micronesia (Federated States of), Kiribati, Kuwait, Lesotho, Marshall Islands, Nauru, Palau, French Polynesia, Tokelau, Tonga, Tuvalu, United States of America, Samoa, South Africa.

***Club 2 (181 countries):***

Afghanistan, Angola, Albania, Andorra, United Arab Emirates, Argentina, Armenia, Antigua and Barbuda, Australia, Austria, Azerbaijan, Burundi, Belgium, Benin, Burkina Faso, Bangladesh, Bulgaria, Bahrain, Bahamas, Bosnia and Herzegovina, Belarus, Belize, Bermuda, Bolivia, Brazil, Barbados, Brunei Darussalam, Bhutan, Central African Republic, Canada, Switzerland, Chile, China, Cote d’Ivoire, Cameroon, DR Congo, Congo, Colombia, Comoros, Cabo Verde, Costa Rica, Cuba, Cyprus, Czech Republic, Germany, Djibouti, Dominica, Denmark, Dominican Republic, Algeria, Ecuador, Eritrea, Spain, Estonia, Ethiopia, Finland, Fiji, France, Gabon, United Kingdom, Georgia, Ghana, Guinea, Gambia, Guinea Bissau, Equatorial Guinea, Greece, Grenada, Greenland, Guatemala, Guyana, China (Hong Kong SAR), Honduras, Croatia, Haiti, Hungary, Indonesia, India, Ireland, Iran, Iraq, Iceland, Israel, Italy, Jamaica, Jordan, Japan, Kazakhstan, Kenya, Kyrgyzstan, Cambodia, Saint Kitts and Nevis, South Korea, Lao PDR, Lebanon, Liberia, Libya, Saint Lucia, Sri Lanka, Lithuania, Luxembourg, Latvia, Morocco, Moldova, Madagascar, Maldives, Mexico, Macedonia (TFYR), Mali, Malta, Myanmar, Montenegro, Mongolia, Mozambique, Mauritania, Mauritius, Malawi, Malaysia, Namibia, Niger, Nigeria, Nicaragua, The Netherlands, Norway, Nepal, New Zealand, Oman, Pakistan, Panama, Peru, Philippines, Papua New Guinea, Poland, Puerto Rico, North Korea, Portugal, Paraguay, Occupied Palestinian Territory, Qatar, Romania, Russian Federation, Rwanda, Saudi Arabia, Sudan, Senegal, Singapore, Solomon Islands, Sierra Leone, El Salvador, Somalia, Serbia, Sao Tome and Principe, Suriname, Slovakia, Slovenia, Sweden, Swaziland, Seychelles, Syrian Arab Republic, Chad, Togo, Thailand, Tajikistan, Turkmenistan, Timor-Leste, Trinidad and Tobago, Tunisia, Turkey, Taiwan, Tanzania, Uganda, Ukraine, Uruguay, Uzbekistan, Saint Vincent and the Grenadines, Venezuela, Viet Nam, Vanuatu, Yemen, Zambia, Zimbabwe.
